# Optimization of Phenolic and Flavonoid Extraction From Bee Bread Using Response Surface Methodology and Central Composite Design, and Evaluation of Antimicrobial Activity

**DOI:** 10.1002/fsn3.71723

**Published:** 2026-04-10

**Authors:** Nilay Keyvan, Muhammet Mükerrem Kaya, Melike Sultan Demirağ, Murat Bayezit, Hidayet Tutun, Hatice Ahu Kahraman, Erhan Keyvan

**Affiliations:** ^1^ Department of Food Hygiene and Technology, Faculty of Veterinary Medicine Burdur Mehmet Akif Ersoy University Burdur Türkiye; ^2^ Department of Pharmacology and Toxicology, Faculty of Veterinary Medicine Burdur Mehmet Akif Ersoy University Burdur Türkiye

**Keywords:** antimicrobial activity, antioxidant activity, bee bread, response surface methodology

## Abstract

Bee bread is a remarkable apicultural product recognized for its abundant proteins, minerals, vitamins, and phenolic compounds, which enhance its notable biological activity. This study aimed to optimize the extraction conditions to maximize the total phenolic content (TPC), total flavonoid content (TFC), and antioxidant capacity (DPPH) with conventional extraction of bee bread using response surface methodology (RSM) with Central Composite Design (CCD). The phenolic profile of the optimal extract was evaluated by high‐performance liquid chromatography (HPLC). Antibacterial activity of the optimal extract was evaluated against *Staphylococcus aureus, Escherichia coli*, and *Salmonella* Enteritidis by using the microdilution method. The optimal conditions were determined to be 30°C temperature, 40 mL/g solvent–solid ratio, 66.729% solvent concentration, and 19.996 h time. The TPC, TFC, and DPPH results of the optimal extract were found to be 26.860 ± 1.08 mg GAE/g, 2.300 ± 0.46 mg QE/g, and 3.043 ± 0.26 mg AAE/g, respectively. HPLC analysis showed that quercetin, kaempferol, and hesperidin are the main phenolic compounds of the optimal extract. The optimal extract had minimum inhibition concentration (MIC) values of 12.5 mg/mL against *S* . Enteritidis, 25 mg/mL against *E. coli* , and > 25 mg/mL against *S. aureus* . In conclusion, this study demonstrated that the recovery of antioxidant and phenolic compounds from bee bread can be significantly enhanced by RSM.

## Graphical abstract

This study successfully optimized the extraction parameters for Turkish bee bread using Response Surface Methodology (RSM) with a Central Composite Design (CCD). Under optimized conditions (30°C, 40 mL/g ratio, 66.7% ethanol, 20 h), the recovery of total phenolics (26.86 mg GAE/g) and flavonoids was maximized. HPLC‐DAD characterization identified Quercetin, Kaempferol, and Hesperidin as the primary bioactive markers. The resulting optimal extract demonstrated potent antibacterial efficacy, particularly against *Salmonella Enteritidis* with a Minimum Inhibitory Concentration (MIC) of 12.5 mg/mL, validating the effectiveness of the multivariate optimization approach for functional food development.

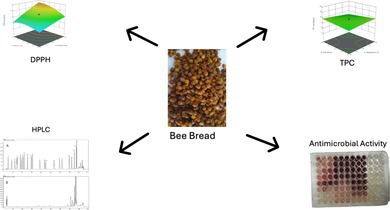

## 1 Introduction

Bee bread is mixed with pollen, honey, and bee saliva and packaged in honeycomb cells. Bee bread undergoes a fermentation process in the cells owing to gland secretion, microorganism and environmental conditions and worker bees use the fermented bee bread to feed the larvae and young bees to create royal jelly (Sobral et al. 2017). Bee bread contains varying amounts of carbohydrates, fatty acids, proteins, vitamin and minerals depending on its botanical origin (Bakour et al. 2019; Kaplan et al. 2019). The protein and soluble sugar of bee bread have displayed a digestible profile into gastrointestinal digestive system (Aylanc et al. 2023). Also, bee bread has a wide variety of other bioactive compounds, such as kaempferol, rutin, quercetin, luteolin, and rosmarinic acid, according to the findings of a number of research studies that have been conducted on the subject of the chemical composition of bee bread (Kaplan et al. 2019; Bakour et al. 2019, 2022; Ćirić et al. 2022). The polyphenols contained in bee bread are responsible for the antioxidant, antibacterial, antifungal, anti‐inflammatory and anticancer activities (Bakour et al. 2022). In addition to these effects, bee bread is known to have effects such as improving the immune system and facilitating digestion (Mărgăoan et al. 2019, 2020).

Due to its important biological effects, bee bread is considered a nutritional supplement, and many studies have been conducted on maximizing biological activity from bee bread and developing functional food or supplement products (Othman et al. 2019; Yıkmış et al. 2024; Keyvan and Yurdakul 2024). Recent research has also revealed how well diverse natural plant extracts and their active chemicals work to improve antioxidant and antibacterial activity in different biological models (Elzaiat et al. 2024; Balgoon and Alghamdi 2024; Luecha et al. 2024; Hammoud et al. 2025). Response surface methodology (RSM) extraction processes are widely used in research aimed at maximizing the antioxidant capacity and biologically active components such as total phenolic (TPC) or total flavonoid content (TFC) (Luecha et al. 2024). This approach determines the optimum process conditions, thus increasing both efficiency and providing a scientific basis for functional product development (Dranca et al. 2020a; Yıkmış et al. 2024). RSM produces significant effects on the response variables antioxidant and TPC from the independent variables (temperature, solvent component). The extraction technique substantially influences the yield and bioactivity of bee bread. Ethanol and water are often utilized solvents, with ethanol typically exhibiting superior antibacterial efficacy (Chunling et al. 2009). The selection of solvent and extraction parameters can influence the phenolic content and, therefore, the antioxidant and antibacterial characteristics of the extracts (Chunling et al. 2009; Pełka et al. 2021). Ethanol extracts provide superior antibacterial activity relative to water extracts, especially against *Bacillus subtilis* and *Staphylococcus aureus* (Chunling et al. 2009). Therefore, this study aimed to optimize the extraction conditions to maximize the total phenolic content, total flavonoid content and antioxidant capacity of bee bread using RSM. Additionally, the antibacterial activity of the optimum extract against some food pathogens was also evaluated to holistically evaluate the success of the multivariate optimization strategy.

## 2 Materials and Methods

### 2.1 Preparation of Beebread Samples

Bee bread samples were obtained from apiaries in October 2022 in Ankara province of Turkiye. The samples have been lyophilized by using a freeze dryer (Martin Christ, Alpha 1–2 LD plus, Germany) and kept in a sealed bottle in the dark at −20°C. The dried samples were ground to powder via a commercial blender prior to their analysis.

### 2.2 Experimental Design for the Response Surface Procedure

RSM was used to establish the optimal conditions for antiradical activity, TPC, and TFC extraction from Bee bread. The optimization process was assessed by using the Design Expert v22.0 (Stat Ease Inc, Minneapolis, MN, USA) software. A five‐level, four‐factor central composite design (CCD) was used to identify relationships existing between the response functions and independent variables. Temperature (X_1_, 20°C–60°C), Solvent‐Solid ratio (X_2_, 10–50 mL/g), solvent concentration (X_3_, 0–100%), and extraction time (X_4_, 2–26 h) were the independent variables and ranges studied. The responses (dependent variables) of design were measured TPC, DPPH radical scavenging ability assay (DPPH), TFC. To obtain optimum conditions, TPC, TFC and antioxidant capacity were entered into the model as mg GAE/g, mg QE/g and mg AAE/g dry bee bread, respectively. Extraction was performed under optimum conditions predicted by the model. Experimental results and estimated values were compared. The optimum extract was lyophilized to obtain dry extract. HPLC analyses were performed on this dry optimum extract.

The experimental data were fitted using a generalized quadratic polynomial model to predict response variables. A second‐order polynomial model, presented in Equation (1), was applied to predict the response variable as a function of the studied independent factors.

$$Y=\beta_{0}+\sum_{i=1}^{k}\text{βiXi}+\sum_{i=1}^{k}\text{βiiX}i^{2}+\sum_{i=1}^{k-1}\sum_{j=i+1}^{k}\text{βijXiXj}+\varepsilon$$

where Y_k_ is the predicted response variable, β_0_ is the regression coefficient for intercept, and β_i_, β_ii_, and β_ij_ are the linear, quadratic, and interaction regression coefficients, respectively, X_i_, X_ii_, and X_ji_ represent the independent variables. An analysis of variance (ANOVA) with a 95% confidence level was performed to assess the effect of each factor on the predicted model on the response variable.

Each variable to be optimized was coded at three levels −α, −1, 0, +1, +α Table 1 shows the values of independent process variables considered in the experimental design, which included 30 extraction runs with five replicates of central points assigned based on CCD.

**TABLE 1:** Levels of experimental variables.

Factors	Symbols	Coded levels				
		−α	−1	0	1	+α
Temperature	**X**_**1**_	20	30	40	50	60
Solvent‐solid ratio	**X**_**2**_	10	20	30	40	50
Solvent concentration	**X**_**3**_	0	25	50	75	100
Extraction time	**X**_**4**_	2	8	14	20	26

### 2.3 Solvent Extraction

Solvent extraction was performed in a temperature‐controlled water bath (Nuve, Turkiye). The independent variables used in the experimental design are given in Table 1. The solvent used for the extraction was ethanol. The experimental design was created using Design‐Expert v22.0 software and a CCD matrix containing a total of 30 conditions was obtained (Table 2). Lyophilized bee bread (1 g) was placed in 50 mL closed borosilicate glass bottles. Each extraction was performed under solvent extraction conditions according to the experimental design. After extraction, the mixture was rapidly cooled on ice and was centrifuged at 3000 rpm for 10 min, and then the extract was filtered and stored at −20°C.

**TABLE 2:** Experimental design and results.

RUN	X_1_	X_2_	X_3_	X_4_	Y (Actual response values)		
	Temperature	Solvent‐solid ratio	Solvent concentration	Extraction time	TPC	TFC	DPPH
	°C	mL/g	%	Hour	mg GAE/g	mg QE/g	mg AAE/g
**1**	50	20	75	8	19.866	2.439	2.986
**2**	50	20	25	8	18.674	1.925	2.707
**3**	40	30	100	14	16.187	1.815	1.97
**4**	40	30	50	14	14.174	1.544	1.925
**5**	20	30	50	14	11.316	1.713	1.323
**6**	30	20	75	8	10.441	0.87	1.024
**7**	30	20	75	20	9.097	2.264	1.539
**8**	40	10	50	14	25.537	1.997	2.852
**9**	30	20	25	8	15.949	2.196	1.540
**10**	40	50	50	14	19.096	2.229	2.185
**11**	50	40	75	8	11.696	1.803	1.339
**12**	30	20	25	20	15.081	2.016	1.554
**13**	40	30	50	2	15.338	1.788	2.133
**14**	30	40	75	20	18.890	2.273	2.193
**15**	30	40	25	20	18.101	2.28	2.235
**16**	60	30	50	14	18.057	2.537	2.201
**17**	40	30	50	14	17.624	2.164	2.240
**18**	30	40	25	8	17.424	2.049	2.637
**19**	50	40	75	20	18.648	2.303	2.186
**20**	40	30	50	14	12.199	2.679	1.181
**21**	50	40	25	8	15.350	2.069	1.556
**22**	40	30	50	14	18.273	2.311	2.136
**23**	50	20	75	20	17.763	2.302	2.231
**24**	40	30	0	14	17.070	2.106	2.221
**25**	40	30	50	26	13.239	1.682	1.875
**26**	50	20	25	20	18.153	2.232	2.860
**27**	40	30	50	14	18.205	2.341	2.213
**28**	30	40	75	8	12.849	1.941	1.396
**29**	50	40	25	20	18.522	1.716	1.391
**30**	40	30	50	14	15.425	1.929	1.980

*Note:* TPC, TFC, and DPPH values extracts obtained from 30 experiments in bee bread are shown in Table 2. The range of TPC values for bee bread was from 9.097 to 25.537 mg GAE/g bee bread, TFC values ranged from 0.87 to 2.679 mg QE/g dried bee bread, and antioxidant capacity ranged from 1.024 to 2.986 mg AAE/g dried bee bread.

### 2.4 Extract Analysis

#### 2.4.1 TPC

The Folin–Ciocalteu method was used to determine the TPC of bee bread extracts (Zhang et al. 2006; Pełka et al. 2021). Briefly, 80 μL of Folin–Ciocalteu reagent diluted 1:10 with deionized ultrapure water was added to wells containing gallic acid standard solution (dissolved in ethanol, 3.13–400 GAE/mL) or 20 μL of extract. After 5 min. incubation, 60 μL of Sodium carbonate (Na_2_CO_3_) solution (7.5%) was added to the wells. After shaking, 140 μL of ultrapure water was added (to a final volume of 300 μL), and the mixture was incubated in the dark at ambient temperature for 30 min. Color intensity (absorbance at 725 nm) was measured using a microplate reader (MultiskanGo, ThermoScientific). The content of phenolic compounds in the extract is expressed as mg gallic acid equivalents (GAE) per gram of dried bee bread. TPC was quantified using a gallic acid calibration curve *R* ^2^ = 0.9973.

#### 2.4.2 TFC

TFC were determined by the aluminium chloride colorimetric method as described by Sembiring et al. (2018) using quercetin as a standard compound. Briefly, 10 μL of 10% (w/v) aluminium chloride (AlCl_3_) was added to the wells of 96‐well plates containing 50 μL of an extract or quercetin standard solution (3125–200 μg/mL in ethanol). Then, 96% ethanol and 1 M sodium acetate (C_2_H_3_NaO_2_) solutions were added in 150 μL and 10 μL amounts, respectively. The plate was mixed gently and incubated for 40 min. at room temperature in the dark. The plate was read at 415 nm using a microplate reader (MultiskanGo, ThermoScientific). The TFC in extracts is expressed as mg quercetin equivalent (QE) per gram of dried bee bread. TFC was determined using a quercetin calibration curve (*R* ^2^ = 0.9993).

#### 2.4.3 Antiradical Activity

Measurement of 2,2‐Diphenyl‐1‐picrylhydrazil (DPPH) was made according to the method described by Ben Mansour et al. (2016) and Herald et al. (2012) with minor modifications. Briefly, 170 μL of 200 μM methanolic DPPH solution was added to the wells of a 96‐well plate containing 30 μL of extract or ascorbic acid (200.00–3.125 μg/mL) dissolved in ethanol. The mixture was incubated at 25°C for 30 min. in the dark. After incubation, absorbance was measured at 517 nm using a microplate reader (MultiskanGo, ThermoScientific). The antioxidant capacity in the extract is expressed as mg ascorbic acid equivalents (AAE) per gram of dried bee bread. DPPH was expressed using a Trolox calibration curve (*R* ^2^ = 0.9995).

### 2.5 Determination of Polyphenols in Optimal Extract of Bee Bread

The determination of polyphenolic compounds from the lyophilized optimal extract of bee bread was performed by HPLC using an Agilent Eclipse XDB‐C18 (250 × 4.60 mm) 5‐μm column and a chromatograph equipped with a diode array detector (SPD‐M10A, Shimadzu) Standards: gallic acid, protocatechuic acid, catechin, p‐hydroxybenzoic acid, chlorogenic acid, caffeic acid, epicatechin, syringic acid, vanillin, p‐coumaric acid, ferulic acid, sinapinic acid, benzoic acid, o‐coumaric acid, rutin, hesperidin, rosmarinic acid, eriodyctiol, cinnamic acid, quercetin, luteolin, and kaempferol. The extracts were filtered through a polytetrafluoroethylene (PTFE) (0.45 μm) filter and then injected into a 20 μL HPLC system. Phenolic component analyses were performed at the Süleyman Demirel University, Innovative Technologies Application and Research Center (YETEM) as a service procurement. The chromatograms of both the standard mixture and the representative sample presented herein were provided by the laboratory to demonstrate system suitability and retention time confirmation for the analytical batch in which our samples were processed.

### 2.6 Antimicrobial Activity

The antibacterial efficacy of lyophilized optimum extract was evaluated using the microdilution method (CLSI 2016). From the stock culture collection of the Department of Food Hygiene and Technology Laboratory, Burdur Mehmet Akif Ersoy University, strains of *Staphylococcus aureus* (ATCC 25923), *Escherichia coli* (ATCC 29998), and *Salmonella* Enteritidis (ATCC 13076) were transferred on Tryptic Soy Agar (TSA, BK047HA, BİOKAR). The bacterial strains were cultured for 18–24 h at 37°C. Every bacterial cell was adjusted to 0.5 McFarland scale (1–1.5 × 10^8^ CFU/mL) and placed into 0.9% sterile saline buffer. The samples were prepared in Mueller Hinton broth (BK048HA, BIOKAR) and then transferred to the 96 wells of microplates. The serial dilutions of the samples were 25, 12.50, 6.25, 3.12, 1.56, 0.78, 0.39, and 0.19 mg/mL over the course of the experiment. There were twenty microliters of each bacterial inoculum that were placed into each well, and then the plates were incubated at 37°C for twenty‐four h (Keyvan et al. 2022). The minimum inhibitory concentration (MIC) value was determined by adding 20 μL of 2,3,5‐triphenyl‐tetrazolium chloride (1% TTC, Sigma‐Aldrich) to each well for each bacterial species. This was done after the incubation period had been completed. To incubate the plates, they were left at 37°C for three hours. The appearance of a red color shift in those wells indicated the presence of microorganisms that were metabolically active (Karpiński 2019).

### 2.7 Statistics

Content analyses were performed at least twice and results were given as mean ± standard deviation. Optimization with response surface method was performed using Design‐Expert v22.0 program. Pearson correlation was applied using Rstudio software program to determine linear correlation between dependent variables. Significance was accepted as *p* < 0.05 in all statistical tests.

## 3 Results and Discussion

### 3.1 Optimization of Extraction Conditions

This study used an experimental CCD to assess the effect of four factors on the TPC, TFC, and antioxidant activity (DPPH) of bee bread: temperature, solvent‐solid ratio, solvent concentration, and extraction time.

### 3.2 Fitting the Model

The significance and adequacy of the RSM quadratic model were assessed using data obtained from the CCD for all response variables. The data in Table 3 were analyzed by multiple regression fitting and three quadratic multinomial equations describing the relationship between the three variables and DPPH, TPC, and TFC were obtained. The mathematical models that represent the link between the significant independent variables and the response variables are shown in Equations.

$$\begin{array}{c} Y_{\mathrm{TPC}}=+18,29–0,0979\;X_{1}+1,89\;X_{2}+1,17\;X_{3}\\ \;-0,1948\;X_{4}–0,6420\;X_{1}X_{2}-1,48\;X_{1}X_{3}\\ \;-1,34\;X_{1}X_{4}+1,16\;X_{2}X_{3}+1,45\;X_{2}X_{4}\\ \;+0,2517\;X_{3}X_{4}–0,2597\;{X_{1}}^{2}–0,6425\;{X_{2}}^{2}\\ \;-1,57\;{X_{3}}^{2}–0,0433\;{X_{4}}^{2} \end{array}$$

$$\begin{array}{c} Y_{\mathrm{TFC}}=+2,28+0,0815\;X_{1}-0,0325\;X_{2}\\ \;+0,1545\;X_{3}+0,0388\;X_{4}\;0,0039\;X_{1}X_{2}\\ \;+0,0233\;X_{1}X_{3}+0,0281\;X_{1}X_{4}-0,0239\;X_{2}X_{3}\\ \;+0,0171\;X_{2}X_{4}-0,0021\;X_{3}X_{4}-0,0130\;{X_{1}}^{2}\\ \;+0,0464\;{X_{2}}^{2}-0,2916\;{X_{3}}^{2}-0,0270\;{X_{4}}^{2} \end{array}$$

$$\begin{array}{c} Y_{\text{DPPH}}=+2,20+0,0108\;X_{1}+0,4578\;X_{2}\\ \;+0,2350\;X_{3}+0,0163\;X_{4}+0,0070\;X_{1}X_{2}\\ \;-0,0304\;X_{1}X_{3}-0,0095\;X_{1}X_{4}+0,1474\;X_{2}X_{3}\\ \;+0,0200\;X_{2}X_{4}+0,0274\;X_{3}X_{4}-0,0105\;{X_{1}}^{2}\\ \;-0,423\;{X_{2}}^{2}-0,1946\;{X_{3}}^{2}-0,0112\;{X_{4}}^{2} \end{array}$$

**TABLE 3:** The analysis of variance (ANOVA) of the quadratic models.

Response value	Source	Sum of squares	df	Mean square	*f*	*p*	Significance
**TPC**	Model	321.27	14	22.95	36.25	< 0.0001	significant
	Residual	9.50	15	0.6330			
	Lack of fit	8.52	10	0.8520	4.37	0.0586	not significant
	Pure error	0.9748	5	0.1950			
	Cor total	330.77	29				
	*R* ^2^	0.9713					
	*R* ^2^ adj	0.9445					
	*R* ^2^ pre	0.8474					
	C.V. %	4.89					
**TFC**	Model	3.39	14	0.2420	21.70	< 0.0001	significant
	Residual	0.1673	15	0.0112			
	Lack of fit	0.1486	10	0.0149	3.97	0.0705	not significant
	Pure error	0.0187	5	0.0037			
	Cor total	3.56	29				
	*R* ^2^	0.9529					
	*R* ^2^ adj	0.9090					
	*R* ^2^ pre	0.7517					
	C.V. %	5.15					
**DPPH**	Model	7.81	14	0.5578	114.13	< 0.0001	significant
	Residual	0.0733	15	0.0049			
	Lack of fit	0.0660	10	0.0066	4.50	0.0554	not significant
	Pure error	0.0733	5	0.0015			
	Cor total	7.88	29				
	*R* ^2^	0.9907					
	*R* ^2^ adj	0.9820					
	*R* ^2^ pre	0.9505					
	C.V. %	3.51					

Variance analyses (ANOVA) of experimental results are shown in Table 3. According to the ANOVA results, quadratic regression models with response values of TPC, TFC, and DPPH were subjected and the findings revealed that the three models were highly significant (*p* < 0.0001). The *F* values of the response values were 36.25, 21.70, and 114.13, respectively. The fact that the *F* values for the three variables are significantly high indicates that the experimental variation is explained by the response variables. The Lack of Fit term was also found to be insignificant (*p* > 0.05), showing that the model is adequate for the experimental data. The coefficient of determination *R* ^2^ values for regression model predicted for TPC, TFC, DPPH were 0,9713, 0,9529, and 0,9907 respectively. All coefficients were greater than 0.80, indicating that the data can explain 99% of the three models. The coefficient of variation (CV) values obtained for DPPH, TPC and TFC are 4.89, 5.15, and 3.51, respectively. CV < 10% demonstrates repeatability, which shows that the model is highly reliable and accurate. (Setyaningsih et al. 2023).

### 3.3 Optimization of Extraction Parameters

In this study, it was attempted to optimize the conventional extract process for achieving the maximum yield of TPC, TFC, and DPPH from bee bread. The optimal conditions were 30°C, 40 mL/g, 66.729% and 19.996 h. Extraction was performed under optimum conditions and TPC, DPPH, and TFC values were determined in the obtained extract (Table 4). It was determined that there was no significant difference between the experimental results and the predicted values. The obtained desirability of optimal conditions was 0.846. Ideally, the maximal desirability was used at the maximum concentrations of dependent variables. The accuracy of the predicted values was evaluated through validation experiments and confirmed by showing that the error rates remained below 10% (Ataya et al. 2025).

**TABLE 4:** The estimated coefficients of quadratic models and their statistical significance.

Model parameter	TPC		TFC		DPPH	
	Coefficient	*p*	Coefficient	*p*	Coefficient	*p*
**Intercept**	18.29		2.28		2.20	
**X**_**1**_	−0.0979	0.5555	0.0815	0.0018	0.0108	0.4595
**X**_**2**_	1.89	< 0.0001	−0.0325	0.1520	0.4578	< 0.0001
**X**_**3**_	1.17	< 0.0001	0.1545	< 0.0001	0.2350	< 0.0001
**X**_**4**_	−0.1948	0.2490	0.0388	0.0921	0.0163	0.2703
**X**_**1**_ **X**_**2**_	−0.6420	0.0056	0.0039	0.8834	0.0070	0.6944
**X**_**1**_ **X**_**3**_	−1.48	< 0.0001	0.0233	0.3912	−0.0304	0.1027
**X**_**1**_ **X**_**4**_	−1.34	< 0.0001	0.0281	0.3047	−0.0095	0.5947
**X**_**2**_ **X**_**3**_	1.16	< 0.0001	−0.0239	0.3790	0.1474	< 0.0001
**X**_**2**_ **X**_**4**_	1.45	< 0.0001	0.0171	0.5279	0.0200	0.2704
**X**_**3**_ **X**_**4**_	0.2517	0.2250	−0.0021	0.9388	0.0274	0.1381
**X**_**1**_^**2**^	−0.2597	0.1080	−0.0130	0.5286	−0.0105	0.4455
**X**_**2**_^**2**^	−0.6425	0.0007	0.0464	0.0363	−0.0423	0.0063
**X**_**3**_^**2**^	−1.57	< 0.0001	−0.2916	< 0.0001	−0.1946	< 0.0001
**X**_**4**_^**2**^	−0.0433	0.7797	−0.0270	0.2004	−0.0112	0.4143

### 3.4 Antioxidant Activity

**FIGURE 1:**
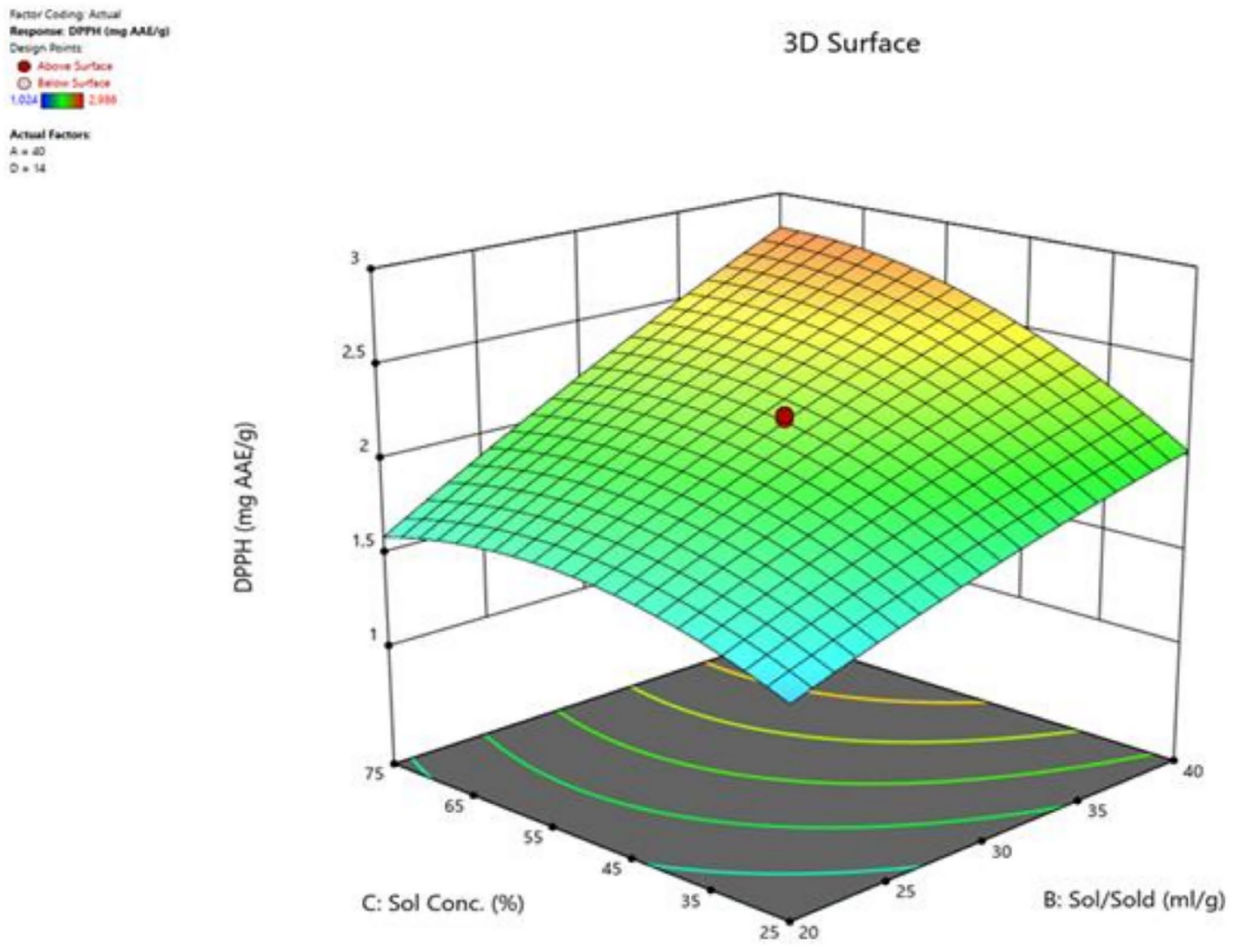
Three‐dimensional (3d) response surface plots for the effect of solvent‐solid ratio–solvent concentration on DPPH.

Multiple linear regressions were used to determine the regression coefficients for dependent variables as shown in Table 5. In terms of linear terms, Solvent/Solid Ratio (X_2_) and Solvent Concentration (X_3_) have positive linear effects. Both variables are significant for DPPH. As the Solvent volume increases, the dissolution of phenolic compounds increases, but in our study, it was observed that after the optimum point, the increase in solvent volume (X_2_^2^) decreased the antioxidant capacity because it decreased the compound concentration. Solvent composition also affects the dissolution of polyphenols; in our study, it shows that more or less ethanol ratio (X_3_^2^) negatively affects the efficiency. Only the interaction effect of Solvent/Solid Ratio–Solvent Concentration (X_2_X_3_) was found to be significant, and the interaction effects showed a positive effect on the model for DPPH scavenging ability. The quadratic effect of Solvent‐Solid Ratio (X_2_) and Solvent Concentration (X_3_) produced a significant and negative effect on DPPH. Figure 1 shows the three‐dimensional response surface plots depicting the interactions between the independent variables related to the DPPH value extracted using bee bread. The DPPH activity of the bee bread extraction process is shown by the response surface plots of X_2_X_3_ as a function of Solvent‐Solid ratio and solvent concentration. The increase in ethanol and solvent/solid ratio together increased the yield of compounds with antioxidant activity (Figure 1). These results support the necessity of RSM based multivariate optimization to obtain maximum antioxidant capacity from bee bread.

**TABLE 5:** Predicted and experimental values of optimum extract (per gram of dried bee bread).

Dependent variables	Predicted value	Experimental value
TPC (mg GAE/g)	24.589	26.860 ± 1.08
TFC (mg QE/g)	2.135	2.300 ± 0.46
DPPH (mg AAE/g)	2.83	3.043 ± 0.26

Antioxidant capacity of bee bread extract in this study ranged from 1.024–2.986 mg AAE/g dried bee bread (optimum extract = 3.053 mg AAE/g dried bee bread). When the studies in the literature were examined, Bakour et al. (2017) reported that the AAE value in bee bread samples obtained from Morocco was 143.78 ± 11.38 mg/extract. Similarly, in another study conducted by Bakour et al. (2021), the AAE value in Morocco bee bread samples was reported as 65.44 ± 6.34 mg/g extract. In the current study, the antioxidant capacity of the optimal extract of bee bread was found to be quite low compared to the amounts reported in the literature, which may be due to the different geographical origins and environmental conditions of the samples. In addition, it should be taken into account that factors such as plant source, processing conditions, and storage time may also have an effect on these results.

### 3.5 TPC

**FIGURE 2:**
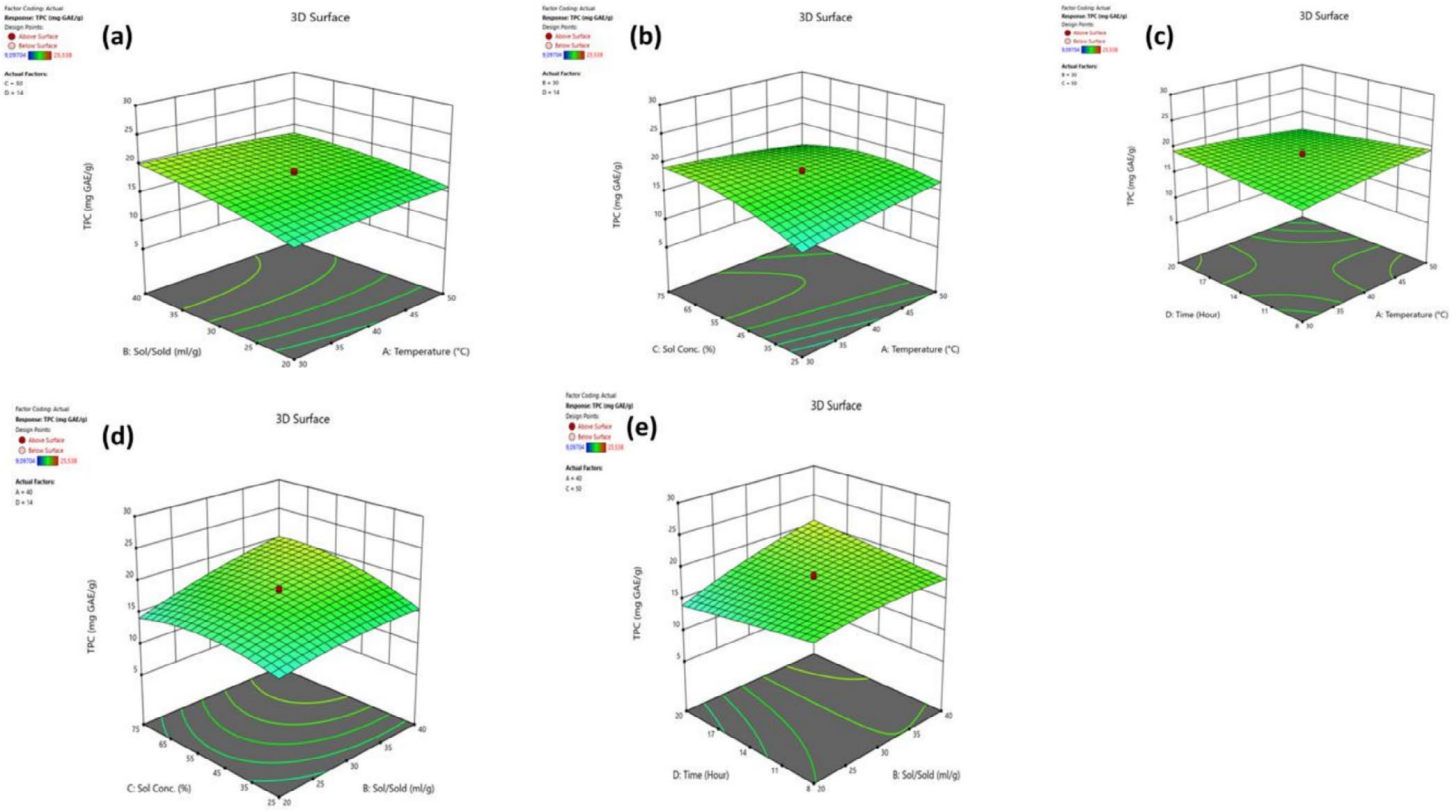
Three‐dimensional (3D) response surface plots for the effect of interaction effects on TPC.

Tables 3, 4 show the coefficients of the independent variables and ANOVA results of TPC values of bee bread extracts. Figure 2 (a, b, c, d, e) shows the three‐dimensional response surface plots illustrating the interactions between the independent variables affecting the TPC value extracted from bee bread. The results show that Solvent‐Solid ratio (X_2_) and Solvent concentration (X_3_) have a statistically significant positive effect on TPC value, whereas temperature (X_1_) and time (X_4_) have a negative effect (statistically insignificant). All quadratic terms (X_12_, X_22_, X_32_ and X_42_) have a negative effect on TPC values, while other quadratic terms except X_12_ and X_42_ produced a significant negative effect on TPC. It was observed that TPC efficiency increased as solvent volume or ethanol ratio increased, but started to decrease after the optimum point. Interaction terms between independent variables, such as X_1_X_2_, X_1_X_3_, X_1_X_4_, X_2_X_4_, X_3_X_4_, have a significant effect on TPC value except X_3_X_4_. Temperature alone did not have a significant effect on TPC yield, but when it increased with all other independent variables, it had a negative effect on TPC yield. Literature reports that higher temperatures and longer exposure times can reduce the diversity and amount of polyphenols in the extract (Vergara‐Salinas et al. 2012; Choulitoudi et al. 2021). Positive interactions between solvent volume and ethanol ratio (X_2_X_3_) and time (X_2_X_4_) may have increased the extraction of phenolic compounds and high temperature may have destroyed the increased phenolic compounds. It has been emphasized that high solvent ratio (60%–70%), low extraction temperature (10°C) and long extraction time (24.2 h) are needed to obtain maximum phenolic compound and antioxidant capacity from bee pollen (Kim et al. 2015; Lawag et al. 2021).

In the current study, TPC result was found 9.097 and 25.537 mg GAE/g bee bread samples Can et al. 2024, reported that the TPC contents of bee bread samples vary between 4.393 and 14.917 mg GAE/g dry weight. In another study, the total phenolic values of bee bread samples collected from Ardahan city center and districts ranged from 11.70–18.35 mg GAE/g (Aksoy et al. 2024). Similarly, in the study conducted by Beykaya et al. 2021, it was reported that the values of TPC in the Anatolian bee bread samples were in the range of 11.90–14.77 mg GAE/g. Another report demonstrated that TPC of bee bread from Türkiye ranged between 2.041 ± 0.170 and 3.224 ± 0.006 mg GAE/g bee bread (Kolayli et al. 2024). The TPC values found in this research were generally consistent with those reported in the literature. This suggests that the chemical composition and phenolic content of the analyzed samples may have been largely affected by similar environmental and botanical factors.

### 3.6 TFC

ANOVA results were given in Table 3, 4 for TFC in bee bread extracts, indicating that model terms are a significant effect, remarkable due to values of *p* < 0.05. As it was seen from Table 3 that the two independent variables (X_1_ and X_3_), two quadratic terms (X_2_ and X_3_) significantly affected the TFC value from bee bread, while the interaction effects have no significant effect on TFC value from bee bread extraction. Increasing temperature increased flavonoid release, and as the ethanol content increased, the dissolution of flavonoids increased to a certain point, but it was observed that the solubility of flavonoids decreased at high concentrations. As the amount of ethanol increased, the amount of water‐soluble flavonoids may have decreased. Solvent polarity directly affects the extraction efficiency depending on the structural properties of flavonoids. This supports the fact that both water and ethanol play a role in the solubility of flavonoids and that the optimal mixture is at 50%–70% ethanol level (Shehata et al. 2021; Lin et al. 2021; Iftikhar et al. 2025). Thus, it can be concluded that solvent polarity and temperature should be optimized together in flavonoid extraction.

In the current study, the TFC values obtained during optimization varied between 0.87 and 2.679 mg (2.300 mg in optimum extraction) QE/g dried bee bread. These values were found to be lower compared to some studies reported in the literature, while they were consistent with some studies. The TFC values in bee breads obtained from various regions of Turkey varied between 0.323 and 4.44 mg QE/g bee bread (Kolayli et al. 2024; Can et al. 2024; Mayda et al. 2020). Similarly, Didaras et al. (2021) reported that the TFC value in bee bread samples originating from Greece ranged between 2.56 and 5.49 mg QE/g. Although the TFC values obtained in our study are similar to some studies reported in the literature, they remain relatively low, especially when compared to the higher TFC values reported by Didaras et al. (2021) and Mayda et al. (2020). It is thought that these differences may be due to several basic factors. Firstly, the extraction methods and solvent types used significantly affect the extraction efficiency of flavonoid compounds. Different solvents and extraction conditions may have a direct effect on the TFC results by increasing or decreasing the solubility of flavonoids from the matrix. In addition, the phytogeographic origin of bee bread, its plant source and the feeding habits of bees are among the critical factors determining the phenolic and flavonoid content. Variations in vegetation, climate conditions, soil structure and environmental stress factors can lead to significant differences in the bioactive compound content of bee bread.

### 3.7 Correlation Analyses and Principal Component Analysis (PCA) of Depended Variables

**FIGURE 3:**
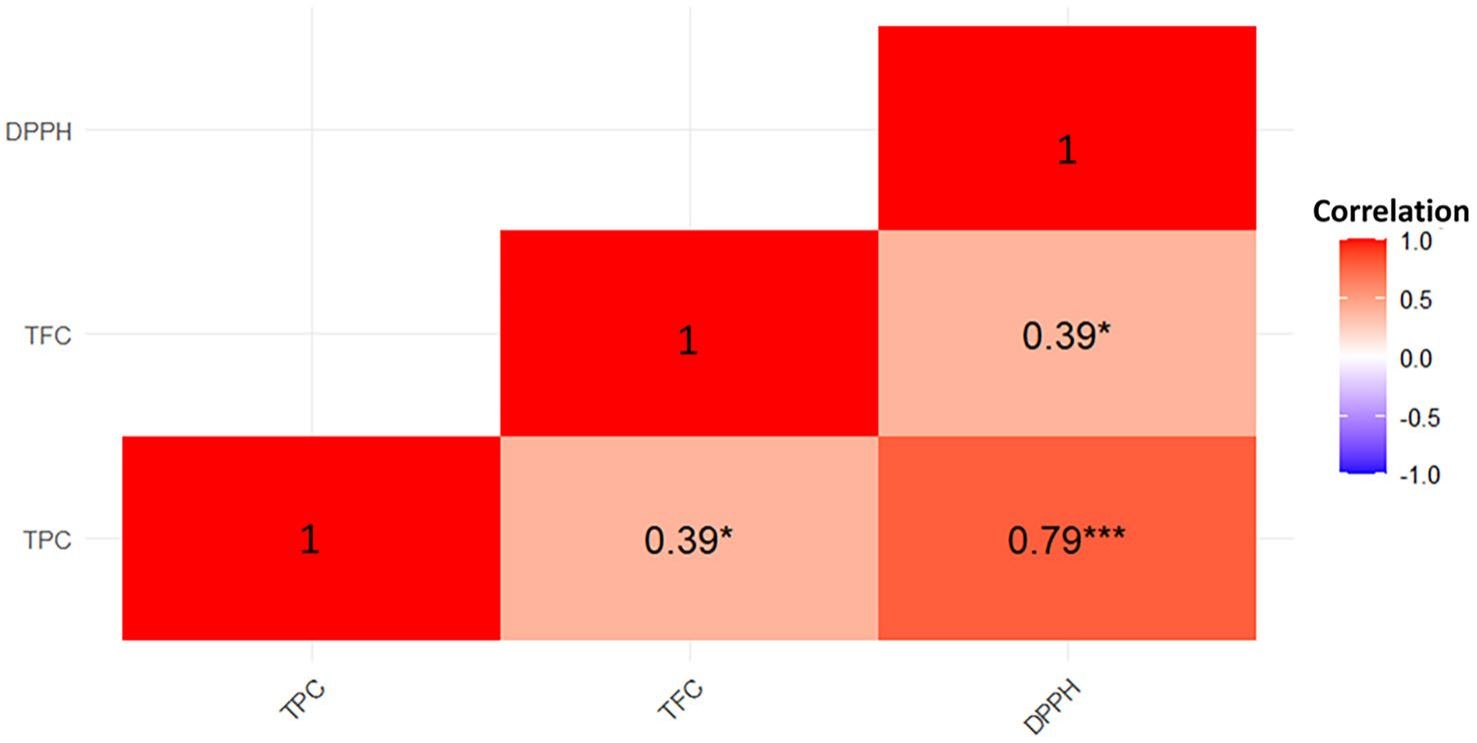
Correlation matrix of dependent variables. *p < 0.05,** *p* < 0.01, ***p < 0.001.

**FIGURE 4:**
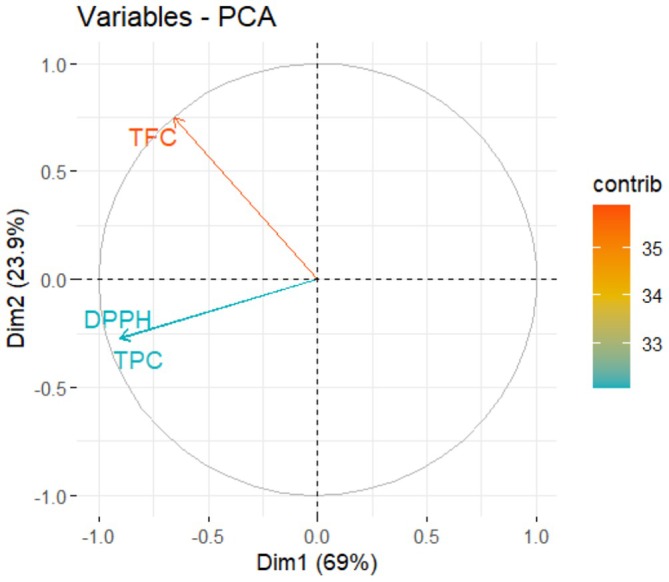
PCA of dependent variables.

The correlation matrix and PCA results of bee bread extract are presented in Figures 3 and 4 respectively. The correlation analysis revealed that there were significant and positive relationships between all dependent variables (TPC, TFC and DPPH). This finding largely coincides with the studies on bee bread in the literature. For example, Aksoy et al. (2024) reported a correlation coefficient of 0.71 between TPC and DPPH, 0.91 between TPC and TFC and 0.73 between TFC and DPPH in their study on bee bread. Similarly, Sawicki et al. (2022) also reported strong positive correlations among TPC, TFC and DPPH parameters in bee bread extracts. The positive correlations obtained in the current study suggest that antioxidant capacity may be directly related to the presence of phenolic and flavonoid compounds (El Guezzane et al. 2024). However, some studies have reached different results than the findings obtained in this study. Can et al. (2024) reported negative correlations between TPC and DPPH and TFC and DPPH at the level of −0.670 and −0.819, respectively, in their study on bee breads collected from different regions of Turkey. Similarly, Didaras et al. (2021) found a negative correlation of −0.586 between TPC and DPPH in a study conducted in Greece. These differences are most likely due to the variability of the content composition of bee bread depending on its botanical and geographical origin, region and environmental conditions Didaras et al. (2021). As a result, the correlation findings obtained in this study are consistent with most literature data and support that the antioxidant capacity of bee bread is closely related to its chemical composition.

Figure 4 presents the results of PCA of the dependent variables from bee bread extracts. Eigenvector values were calculated for all dependent variables and the variance explanation rates were determined. In the PCA analysis, it is considered ideal that the first two components explain more than 70% of the total variance. In this study, the Dim1 (PC1) explains 69.00% and the Dim2 (PC2) explains 23.90% of the variance. These PC together represent 92.90% of the total variance, indicating that the PCA method is suitable for summarizing the multivariate structure of the data of bee bread extracts. As shown in Figure 4, a high level of relationship was observed between the TPC and DPPH variables. These two variables are positioned in the same direction, indicating that they show similar directional variations. On the other hand, the TFC variable is located in a different position in the PCA plane and exhibits a more limited relationship with TPC and DPPH. These shows that TFC has a different distribution pattern compared to the other two dependent variables and has lower variance explanation power.

### 3.8 Polyphenolic Compounds of Optimized Crude Extract

**FIGURE 5:**
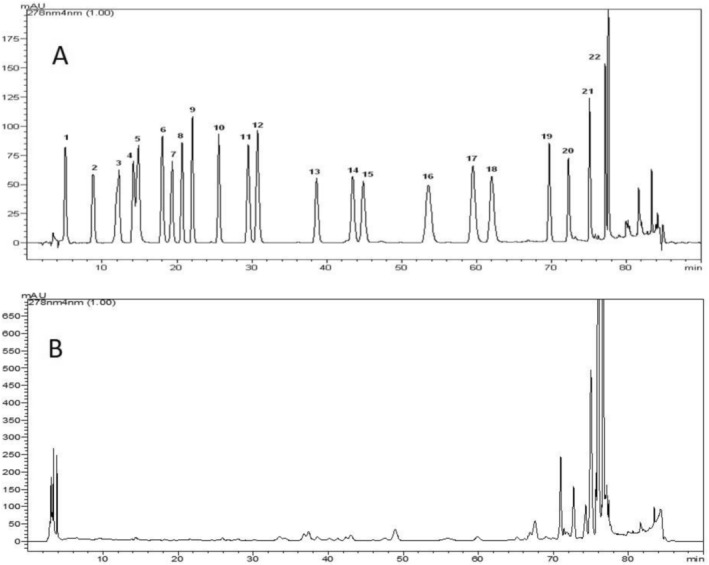
Chromatogram of the optimal extract of bee bread (B) and the standard mixture (A). Peak identification: 1: Gallic acid, 2: Protocatechuic acid, 3: Catechin, 4: *p*‐Hydroxybenzoic acid, 5: Chlorogenic acid, 6: Caffeic acid, 7: Epicatechin, 8: Syringic acid, 9: Vanillin, 10: *p*‐Coumaric acid, 11: Ferulic acid, 12: Sinapinic acid, 13: Benzoic acid, 14: *o*‐Coumaric acid, 15: Rutin, 16: Hesperidin, 17: Rosmarinic acid, 18: Eriodictyol, 19: Cinnamic acid, 20: Quercetin, 21: Luteolin, 22: Kaempferol.

In the current study, catechin, p‐hydroxybenzoic acid, chlorogenic acid, syringic acid, vanillin, ferulic acid, hesperidin, cinnamic acid, quercetin, and kaempferol were detected using HPLC (Table 6). The chromatograms of standard mixture (A) and optimal extract are shown in Figure 5. In a study by Ilie et al. (2024), they used UHPLC–MS/MS analysis to identify gallic acid, caffeic acid, rutin, and epicatechin in bee bread collected from seven regions of Romania. Gallic acid, caffeic acid, epicatechin, and rutin were not detected in our optimal extract in the current study. In the previously mentioned study, epicatechin was not detected in two of the Romanian bee breads. In a study conducted by collecting from various regions of Turkiye, gallic acid, protocatechuic acid, epicatechin, and rutin were not detected in the phenolic content analysis of bee breads using reverse phase‐high performance liquid chromatography‐photodiode array (RP‐HPLC–PDA), and caffeic acid was not detected in 7 out of 15 samples, but p‐hydroxybenzoic acid, chlorogenic acid, ferulic acid, hesperidin, and quercetin were detected in various bee bread samples as in the bee bread in our study. Syringic acid was detected in our study, but it was not detected in any sample in the previously mentioned study (Can et al. 2024). In another study, phenolic profiles were evaluated in bee bread collected from 5 different hives in Turkiye by Liquid chromatography–mass spectrometry/mass spectrometry (LC–MS/MS) and catechin, chlorogenic acid, quercetin, kaempferol, gallic acid, protocatechuic acid, caffeic acid, rutin, and luteolin were detected. Also, syringic acid and sinapinic acid were not detected (Bayram et al. 2021). However, in our study, catechin, chlorogenic acid, quercetin, and kaempferol were detected but gallic acid, protocatechuic acid, caffeic acid, rutin, and luteolin were not detected. In our study, similar to the study mentioned, sinapinic acid was not detected, on the contrary, syringic acid was detected in our study. In the phenolic profile of bee bread collected from Egypt, vanillic acid (20.32 ± 0.02 μg/g), which is the oxidized form of vanillin, cinnamic acid (313.80 ± 0.09 μg/g), and o‐coumaric acid (10.90 ± 0.06 μg/g) were reported to be detected by HPLC (Elsayed et al. 2021). In our study, vanillin (0.2 mg/kg) and cinnamic acid (0.4 mg/kg) were detected, but o‐coumaric acid was not detected. In a study on Romanian bee bread, rosmarinic acid (0.23 mg/L extract) and luteolin (1.17 mg/L extract) were detected by HPLC with DAD (Dranca et al. 2020b). Rosmarinic acid and luteolin were not detected in our study. Studies in the literature have shown that different phenolic compound results can be observed in bee bread samples collected under different geographical conditions, soil, climate, and pollen botanical origins, using different phenolic compound detection methods (Aksoy et al. 2024; De‐Melo et al. 2018; Ilie et al. 2024).

**TABLE 6:** The phenolic compounds of optimal extract of bee bread.

Phenolic compounds	Concentration (μg/g dried extract)
Gallic acid	*
Protocatechuic acid	*
Catechin	1.1
p‐Hydroxy benzoic acid	3.1
Chlorogenic acid	1.1
Caffeic acid	*
Epicatechin	*
Syringic acid	0.4
Vanillin	0.2
Ferulic acid	0.9
Sinapinic acid	*
Benzoic acid	*
o‐coumaric acid	*
Rutin	*
Hesperidin	13.3
Rosmarinic acid	*
Eriodictiol	*
Cinnamic acid	0.4
Quercetin	54.8
Luteolin	*
Kaempferol	31.7

^*^ Not detected.

### 3.9 Antimicrobial Activity

Polyphenolic rich extract contains many phenolic and flavonoid compounds. These compounds not only have antioxidant activity but sometimes also show strong antimicrobial activity. Therefore, testing the antibacterial activity of the extract obtained under optimum conditions is important in order to investigate the functional effect as well as the chemical yield of the extraction. A fermented product of the beehive, bee bread has shown interesting antibacterial action against several bacterial types, including *S.* Enteritidis, *E. coli* , and *S. aureus* . In the current study, minimum inhibitory concentration (MIC) values of bee bread extract were found on *S.* Enteritidis as 12.5 mg/mL, *E. coli* as 25 mg/mL and *S. aureus* as > 25 mg/mL. Extensive antibacterial action against *S. aureus* has been shown from bee bread extracts. Studies show that ethanolic extracts of bee bread show more inhibition capability with MIC values ranging from 2.5%–10% (v/v) against *S. aureus* strains, including methicillin‐resistant *S. aureus* (MRSA) (Pełka et al. 2021). The abundance of bioactive substances including phenolic acids and flavonoids, which abound in bee bread (Suleiman et al. 2021; Keyvan et al. 2023), explains the antibacterial action. In many investigations, bee bread extracts have also shown bactericidal activity, therefore stopping *S. aureus* from proliferating (Urcan et al. 2018; Ilie et al. 2024). Depending on the concentration and extract processing, the extracts have been found to reduce *E. coli* in different degrees of efficacy. For *E. coli* , for example, Malaysian bee bread showed notable antibacterial action with MIC values of about 1.923 μg/mL (Suleiman et al. 2021). Bee bread's potential as a natural antimicrobial agent has also been highlighted by other studies that have verified its antibacterial qualities against *E. coli* (Keyvan et al. 2023; Elsayed et al. 2021). The antimicrobial activity of bee bread extends to *S.* Enteritidis as well. Bee bread extracts have been reported to inhibit the growth of *Salmonella* strains, with some studies showing effective inhibition at low concentrations (Urcan et al. 2023; Ivanišová et al. 2015). In the current study, the antimicrobial activity of bee bread suggests that it may have potential applications in food preservation and as a functional food ingredient. Also, although it showed high TFC and TPC values, it showed low antimicrobial activity. The results show that compounds with strong antioxidant activity in the extract may not always have strong antimicrobial activity.

## 4 Conclusion

Optimization results clearly show the necessity of multivariate optimization in the extraction of bee bread with variable polyphenolic content. The optimum parameters for bee bread extraction were determined to be 30°C, a solvent‐to‐solid ratio of 40 mL/g, an ethanol concentration of 66.7%, and an extraction time of 20 h. Furthermore, antibacterial tests revealed that bee bread extracts exhibited inhibitory activity against both *S. enteritidis* and *E. coli* , with MIC values ranging from 12.5 to 25 mg/mL. Antibacterial results showed that bee bread may have antibacterial activity and extracts with high polyphenolic yield may not have strong antibacterial activity. Selecting antimicrobial activity as the dependent variable in optimization in RSM can improve the extraction efficiency in the direction of functional effect.

## Author Contributions

Nilay Keyvan: Conceptualization, Writing – review and editing, Writing – original draft. Muhammet Mukerrem Kaya: Methodology, Data curation. Melike Sultan Demirag: Methodology. Murat Bayezit: Methodology. Hidayet Tutun: Conceptualization. Hatice Ahu Kahraman: Methodology. Erhan Keyvan: Methodology, Writing – original draft.

## Funding

The authors have nothing to report.

## Conflicts of Interest

The authors declare no conflicts of interest.

## Data Availability

The data that support the findings of this study are available on request from the corresponding author. The data are not publicly available due to privacy or ethical restrictions.
